# Predicting teachers' organizational change cynicism: the roles of school commitment and teaching competence

**DOI:** 10.3389/fpsyg.2026.1777604

**Published:** 2026-07-16

**Authors:** Hamza Öz, Bahadır Gülbahar, Merve Turpçu

**Affiliations:** 1 Yozgat Bozok University, Yozgat, Türkiye; 2 Kirsehir Ahi Evran University, Kırşehir, Türkiye

**Keywords:** education management, organizational change cynicism, school commitment, suppression effect, teaching competence

## Abstract

This study aims to examine the relationships and predictive effects of the subdimensions of teachers' subjective well-being on their perceptions of organizational change cynicism within the framework of positive psychology theory. Using a cross-sectional correlational design, data were collected from 512 teachers and analyzed using correlation and multiple regression analyses. The results showed that school commitment is a strong and significant negative predictor of organizational change cynicism. In contrast, teaching competence, although negatively correlated with cynicism, demonstrated a positive and significant effect in the regression model. This unexpected pattern indicates a suppression effect, suggesting that teaching competence reveals its unique contribution when shared variance with school commitment is controlled. Together, the predictors explained a meaningful proportion of variance in cynicism. The findings highlight the importance of considering both relational attachment and professional evaluation in understanding teachers' responses to organizational change.

## Introduction

Educational systems are increasingly characterized by continuous and multifaceted change driven by policy reforms, technological advancements, and evolving societal expectations. In such contexts, schools are expected to adapt to complex transformation processes that require not only structural adjustments but also the active engagement of teachers, who play a central role in implementing educational change (Fullan, 2007; Hargreaves and Fullan, 2012). However, research indicates that the success of organizational change initiatives depends largely on how teachers perceive and respond to these processes rather than on the formal design of reforms alone (Oreg et al., 2011). These processes may also trigger resistance and change-related cynicism among employees, particularly in complex and uncertain organizational environments (Grama and Todericiu, 2016).

One of the most salient attitudinal responses to organizational change is organizational change cynicism (OCC), which refers to employees' pessimistic beliefs, distrust, and negative expectations regarding the intentions and outcomes of change efforts (Reichers et al., 1997; Wanous et al., 2000). Organizational change cynicism has been associated with a range of undesirable outcomes, including reduced participation and engagement in change processes and increased resistance to innovation, and has also been linked to reduced organizational commitment and increased resistance to chang (Bernerth et al., 2007; Stanley et al., 2005; Toheed et al., 2019). In educational settings, such attitudes may weaken teachers' willingness to engage in reform initiatives and limit the effectiveness and sustainability of school improvement efforts (Oreg et al., 2011).

Given these implications, identifying the antecedents of organizational change cynicism has become an important focus in organizational and educational research. Previous studies have primarily examined organizational-level predictors such as leadership practices, organizational justice, communication quality, and prior change experiences (Armenakis and Bedeian, 1999; Oreg et al., 2011; Vakola, 2014). While these factors offer valuable insights, they do not fully explain why teachers may differ in their responses to change even within similar organizational contexts. This suggests that individual-level professional characteristics and organizational attachment should also be considered in understanding attitudes toward change.

In this regard, school commitment (SC) represents a key organizational construct reflecting teachers' emotional attachment, identification, and sense of belonging to their schools. In educational settings, school commitment has also been associated with job satisfaction and teacher retention (Anog et al., 2024). Rooted in organizational commitment theory (Meyer and Allen, 1991; Meyer et al., 2002), commitment has been widely recognized as a significant determinant of employees' attitudes and behaviors. Teachers with higher levels of commitment tend to demonstrate stronger alignment with organizational goals, greater engagement in institutional processes, and a higher willingness to support change initiatives (Firestone and Pennell, 1993; Somech and Bogler, 2002). Empirical research in educational contexts also suggests that higher levels of commitment are generally associated with lower levels of negative organizational attitudes, such as withdrawal and disengagement (Skaalvik and Skaalvik, 2011). From this perspective, school commitment may function as a protective factor that is generally associated with more constructive and supportive attitudes toward organizational processes and change initiatives.

Another important variable in this context is teaching competence (TC), which refers to teachers' perceived instructional effectiveness, pedagogical knowledge, and professional capability (Shulman, 1987; Tschannen-Moran and Hoy, 2001). Teaching competence has consistently been linked to positive educational outcomes, including higher instructional quality, improved student achievement, and stronger professional self-efficacy (Darling-Hammond, 2017; Blömeke et al., 2015). Accordingly, it is often assumed that more competent teachers are more adaptive and constructive in their responses to organizational processes.

However, research on teacher professionalism suggests that the relationship between competence and organizational attitudes may be more complex than commonly assumed. Highly competent teachers tend to develop stronger professional judgment, critical reflection skills, and increased sensitivity to organizational inconsistencies (Kelchtermans, 2009; Priestley et al., 2015). These characteristics may lead them to interpret organizational practices more critically, particularly when there is a perceived misalignment between institutional policies and professional standards. Such critical evaluations, under certain organizational conditions, may also contribute to less favorable attitudes toward change processes.

Despite the theoretical relevance of both school commitment and teaching competence, the existing literature has largely examined these constructs independently. There remains a need to better understand their combined and differential contributions to organizational change cynicism. In addition, prior research has rarely considered the possibility that relationships among these variables may involve more complex statistical dynamics, such as suppression effects, in which the inclusion of one predictor variable alters the magnitude or direction of another predictor's relationship with the outcome variable (Conger, 1974; MacKinnon et al., 2000). Identifying such patterns is important, as they may reveal relationships that are not evident in simple bivariate analyses.

Addressing these gaps, the present study aims to examine the extent to which school commitment and teaching competence predict teachers' organizational change cynicism. Specifically, the study investigates both the individual and joint contributions of these variables while also considering the possibility of unexpected or counterintuitive relationships, particularly those indicative of suppression effects emerging from their interaction. By doing so, the study seeks to provide a more nuanced understanding of teachers' attitudinal responses to organizational change and to contribute to the literature by integrating organizational and professional perspectives within a single analytical framework.

## Literature review

### Positive psychology theory

Positive psychology theory examines individuals‘ behaviors and experiences not solely through the lens of problems and shortcomings, but within the framework of their internal dynamics, psychological potential, and the capacity they can develop. This perspective aims to explain the psychological processes that enable individuals to maintain their lives functionally and meaningfully in the face of difficulties and uncertainties. Therefore, positive psychology focuses on the protective and empowering elements that allow individuals to adapt despite the difficulties they experience and maintain their psychological integrity in this process (Seligman and Csikszentmihalyi, 2000). The theory considers well-being in its temporal and contextual dimensions, evaluating individuals' satisfaction evaluations regarding past experiences, their levels of expectation and optimism regarding the future, and their intense participation and flow experiences in the present moment within a holistic framework. In addition, the search for meaning, authenticity, and value-based orientations at the individual level, and taking responsibility, cooperation, and positive orientations toward others in a social context are among the fundamental areas of study of positive psychology. Within this multi-layered structure, well-being, based on the individual's subjective experiences, is positioned as one of the central concepts of positive psychology.

In the positive psychology approach, well-being is conceptualized with the PERMA model, developed by Seligman, which is considered a multidimensional structure. The model argues that an individual's well-being cannot be explained by a single psychological element; rather, it arises from the interaction of complementary components such as positive emotions, active participation in life, positive social relationships, attributing meaning to life, and perception of success (Seligman, 2012, 2018; Forgeard et al., 2011). According to the PERMA model, each component offers independent and unique contributions to the individual's psychological functioning, making well-being a holistic yet analytically solvable structure. In human-centered organizations requiring high emotional labor, such as educational institutions, teachers‘ subjective well-being plays a decisive role in shaping organizational attitudes and behaviors beyond individual performance. In this context, positive psychology views well-being not only as an individual emotional state. This approach considers an individual's interactions with their environment, the meaning they attribute to their experiences, and their reactions to changes as a fundamental psychological resource. Particularly in organizational contexts, this approach offers a strong theoretical foundation for understanding how individuals evaluate processes involving uncertainty and change, and their attitudes toward these processes. Therefore, it can be said that teachers' subjective well-being plays an explanatory role in understanding their attitudes, such as adaptability, flexibility, resistance, or cynicism, during organizational change processes.

When evaluated within the context of the PERMA model, it is observed that individuals with high levels of subjective well-being approach organizational change processes in a more flexible, hopeful, and constructive manner; they tend to view change as an opportunity for development and learning rather than a threat. This situation reveals that subjective well-being functions as a protective psychological mechanism against organizational change cynicism. Therefore, from a positive psychology perspective, organizational change cynicism can be considered not only a negative attitude but also a significant indicator of an individual's level of psychological well-being. This research aims to contribute to the psychological foundations of sustainable change processes in educational institutions by examining the relationship between teachers' subjective well-being levels and organizational change cynicism based on positive psychology theory.

### Subjective well-being-SWB

Subjective well-being, one of the most frequently used concepts in the positive psychology literature, is an individual's self-assessment of how positively they perceive their life (Demir, 2022). It is a broader concept encompassing the level of satisfaction and emotional balance experienced by individuals within the framework of their subjective evaluations of their lives (Gencer, 2018; Schimmack et al., 2002). Subjective well-being reveals the extent to which an individual finds their life meaningful and how they evaluate their life satisfaction (Gencer, 2018).

Research shows that high levels of subjective well-being strengthen individuals‘ social relationships (Diener and Biswas-Diener, 2011; Liao and Li, 2025), increase their economic and professional success (Graham and Pettinato, 2002), improve their creativity and productivity (Staw et al., 1994) and have positive effects on physical health and life expectancy (Røysamb et al., 2003). Therefore, subjective well-being is not only an individual emotional state but also a strong predictor of life satisfaction (Myers and Diener, 1995). In the context of education, the Organisation for Economic Co-operation and Development's (OECD, 2017) report on teacher subjective well-being defines it as a multidimensional construct encompassing individual assessments of the quality of professional life, job satisfaction, perceived meaningfulness, and professional competence. Teachers' subjective well-being is critically important not only for their psychological health but also for student success, the quality of the school climate, and the sustainability of educational policies (Van Veen et al., 2005). In the study conducted by Ergün and Sezgin Nartgün (2017), teacher subjective well-being consists of two sub-dimensions: school commitment and teaching competence. While the sub-dimension scores individually define well-being in the area they measure, the total scale score corresponds to total subjective well-being. The school commitment dimension is considered as feeling supported by individuals in the school and establishing good relationships, while the teaching competence dimension is considered as the individual's ability to evaluate their own teaching as effective and demand oriented.

Subjective well-being is widely conceptualized as a multidimensional construct including life satisfaction, positive affect, and the absence of negative affect (Diener et al., 1999). Recent studies emphasize that subjective well-being functions not only as an outcome variable but also as a psychological resource shaping professional attitudes and behaviors (Hascher and Waber, 2021; Xie et al., 2025). Furthermore, contemporary research highlights that subjective well-being functions as a psychological resource that supports teachers' engagement, resilience, and adaptive functioning in demanding educational environments (Diener et al., 2018).

### Organizational change cynicism

Educational organizations operate in increasingly complex and dynamic environments shaped by policy reforms, accountability demands, and continuous structural transformation. In such contexts, organizational change is not a one-time event but an ongoing process requiring sustained adaptation. However, research suggests that the effectiveness of change initiatives depends not only on structural or managerial decisions but also on how organizational members interpret and respond to these processes (Oreg et al., 2011). Among these responses, organizational change cynicism (OCC) has emerged as a key construct for understanding skepticism, disengagement, and resistance toward reform.

Organizational change cynicism refers to a pessimistic attitude toward change initiatives, characterized by skepticism about the likelihood of success and doubts regarding the sincerity or competence of those responsible for implementing change (Reichers et al., 1997; Wanous et al., 2000). Recent studies further suggest that cynicism about organizational change may be multidimensional, with different dimensions having distinct implications for employee outcomes (Gupta and Mishra, 2024). Unlike general organizational cynicism, which reflects a broader negative orientation toward the organization, OCC specifically targets change processes and reform efforts. It can therefore be understood as a context-specific evaluative stance shaped by prior experiences, perceived credibility of leadership, and expectations about the outcomes of change.

The literature conceptualizes cynicism as a multidimensional construct involving cognitive, affective, and behavioral components (Dean et al., 1998). Individuals with higher levels of cynicism tend to question the intentions behind change, experience negative emotions such as distrust or frustration, and show reduced willingness to engage in implementation processes (Bommer et al., 2005; Stanley et al., 2005). In organizational contexts, such attitudes are generally associated with lower engagement in change initiatives and increased resistance to innovation (Bernerth et al., 2007).

A range of antecedents of OCC has been identified in prior research, including ineffective leadership, inadequate communication, perceived injustice, breaches of trust, and repeated exposure to unsuccessful change efforts (Armenakis and Bedeian, 1999; Johnson and O'Leary-Kelly, 2003; Oreg et al., 2011; Vakola, 2014). Although these findings are largely derived from general organizational research, they may provide a useful framework for interpreting teachers' responses to change. In educational settings, teachers act as key agents of reform implementation; therefore, their interpretations of change processes may play a critical role in shaping the sustainability and effectiveness of school-level transformation efforts.

### School commitment and organizational change cynicism

One of the central variables that may shape teachers' responses to organizational change is school commitment. Commitment is commonly defined as a psychological bond between the individual and the organization, reflected in emotional attachment, identification with organizational goals, and a willingness to remain involved in organizational processes (Meyer and Allen, 1991; Meyer et al., 2002). In school contexts, this construct refers to teachers' sense of belonging to their institution and their alignment with its values and objectives.

Teachers with higher levels of commitment tend to demonstrate greater engagement in school activities, stronger participation in organizational processes, and a higher willingness to contribute to institutional goals (Firestone and Pennell, 1993; Somech and Bogler, 2002). This is consistent with recent evidence suggesting that teachers' organizational commitment remains a critical asset in adapting to evolving education reforms (Xu and Pang, 2024). The broader organizational literature also suggests that commitment is generally associated with more constructive and supportive attitudes toward organizational functioning, as well as lower levels of withdrawal and disengagement (Meyer et al., 2002). In educational research, similar patterns have been observed, with higher commitment being associated with more stable professional engagement and lower tendencies toward withdrawal-related attitudes (Skaalvik and Skaalvik, 2011).

Although the direct relationship between school commitment and organizational change cynicism has not always been examined explicitly, existing theoretical and empirical work suggests that stronger commitment may be associated with less negative interpretations of organizational processes. Teachers who feel more connected to their schools may be more likely to interpret change initiatives as aligned with collective goals and may therefore approach such processes in a more constructive and supportive manner. Conversely, lower levels of commitment may be associated with more detached or skeptical responses to change.

Empirical findings also support the relevance of school commitment for school-related attitudes and outcomes. In educational settings, higher school commitment has been associated with job satisfaction and teacher retention (Anog et al., 2024), while recent evidence also suggests that teachers' organizational commitment remains an important asset in adapting to ongoing educational reforms (Xu and Pang, 2024). In parallel, research on organizational cynicism has shown that cynical attitudes are linked to reduced commitment and stronger resistance to change (Toheed et al., 2019). Taken together, these findings provide an empirical basis for expecting an inverse relationship between school commitment and organizational change cynicism.

### Teaching competence and organizational change cynicism

Another important factor in understanding teachers' responses to organizational change is teaching competence. Teaching competence refers to teachers' perceptions of their instructional effectiveness, pedagogical knowledge, and ability to manage teaching and learning processes (Shulman, 1987; Tschannen-Moran and Hoy, 2001). Recent work also conceptualizes teaching competence as a multidimensional construct involving integrated knowledge, skills, motivations, and attitudes that support professional performance (Mara and Morar, 2023). It encompasses both knowledge-based and practice-oriented dimensions of professional performance.

The literature generally associates teaching competence with positive educational outcomes. In addition, pedagogical competence has been described as a broad construct involving meta-competencies, professional competencies, and practical competencies (Ranta et al., 2023). Competent teachers are more likely to deliver effective instruction, support student learning, and demonstrate stronger professional self-efficacy (Darling-Hammond, 2017; Blömeke et al., 2015). From this perspective, teaching competence is often conceptualized as a professional resource that may facilitate adaptive responses to organizational demands.

However, research on teacher professionalism suggests that competence may also be associated with increased critical reflection and professional judgment. In this context, organizational commitment and teacher professionalism have been found to be closely related in supporting educational quality (Aprilia et al., 2024). Teachers with higher levels of competence often develop more refined criteria for evaluating instructional practices and organizational processes (Kelchtermans, 2009; Priestley et al., 2015). As a result, they may be more sensitive to inconsistencies between institutional expectations and classroom realities. This sensitivity reflects a more evaluative stance rather than a purely adaptive orientation.

Accordingly, teaching competence may be associated with different types of responses depending on contextual conditions. While competence may support adaptive functioning, it may also be associated with more critical evaluations of change initiatives, particularly when such initiatives are perceived as insufficiently aligned with professional standards. Under certain organizational conditions, these critical evaluations may contribute to more critical or less favorable evaluations of change processes, without necessarily implying direct resistance or cynicism.

Prior research also indicates that teaching and pedagogical competence are multidimensional constructs involving integrated knowledge, skills, motivations, attitudes, and evaluative judgment (Mara and Morar, 2023; Ranta et al., 2023). At the same time, the literature on teacher professionalism shows that stronger professional judgment may increase teachers' sensitivity to instructional and organizational inconsistencies (Kelchtermans, 2009; Priestley et al., 2015). Although previous research has generally associated competence with positive professional functioning, these findings also suggest that more competent teachers may evaluate organizational practices more critically under certain conditions. This body of research provides an empirical and theoretical basis for examining the relationship between teaching competence and organizational change cynicism.

### The joint role of school commitment and teaching competence

Although school commitment and teaching competence are both relevant to teachers' organizational attitudes, they represent distinct dimensions of professional experience. School commitment reflects relational attachment to the organization, whereas teaching competence reflects confidence in professional capability. These dimensions may influence attitudes toward change through different psychological mechanisms.

Teachers with higher levels of commitment may interpret change in terms of organizational belonging and shared goals, while teachers with higher competence may evaluate change in terms of professional standards and instructional relevance. As a result, these variables may not always operate in the same direction. Examining them together is therefore important for capturing the complexity of teachers' responses to organizational change.

Despite this relevance, the literature has rarely examined the combined effects of school commitment and teaching competence on organizational change cynicism. This gap limits the ability to identify more nuanced patterns of relationships that may emerge when these variables are considered simultaneously.

Existing research nevertheless suggests that both variables are relevant for understanding teachers' school-related attitudes. School commitment has been associated with engagement, satisfaction, and retention in educational settings (Anog et al., 2024; Xu and Pang, 2024), whereas recent research on cynicism about organizational change has emphasized its potentially multidimensional structure and differentiated outcome patterns (Gupta and Mishra, 2024). These findings support the examination of school commitment and teaching competence not only separately but also jointly in predicting organizational change cynicism.

### Suppression effects and hypothesis development

In addition to their conceptual distinction, school commitment and teaching competence may also interact at a statistical level. In regression analysis, such interactions may take the form of suppression effects, where the inclusion of one predictor clarifies or alters the effect of another predictor on the outcome variable (Conger, 1974; Friedman and Wall, 2005; MacKinnon et al., 2000). Suppression occurs when shared variance between predictors obscures their unique contributions, and controlling for this shared variance reveals relationships that are not apparent in simple bivariate analyses.

In the present context, school commitment and teaching competence are conceptually related yet distinct constructs. When examined together, school commitment may capture the relational and affective dimensions of teachers' organizational experiences, whereas teaching competence may reflect a more evaluative and professional dimension. Controlling for their shared variance may therefore reveal the unique contribution of teaching competence in a way that differs from its simple association with organizational change cynicism.

Based on this framework, school commitment is expected to be negatively associated with organizational change cynicism, as stronger attachment to the school may be linked to more constructive interpretations of change. Teaching competence, while often conceptualized as a positive professional resource, may demonstrate a more complex pattern of association when examined alongside school commitment. Accordingly, examining both variables simultaneously is necessary for developing a more comprehensive understanding of teachers' responses to organizational change.

Based on this framework, the following hypotheses are proposed:

H1: Teachers' school commitment negatively and significantly predicts organizational change cynicism.

H2: Teachers' teaching competence negatively and significantly predicts organizational change cynicism.

H3: School commitment and teaching competence jointly and significantly predict organizational change cynicism.

**Figure 1:**
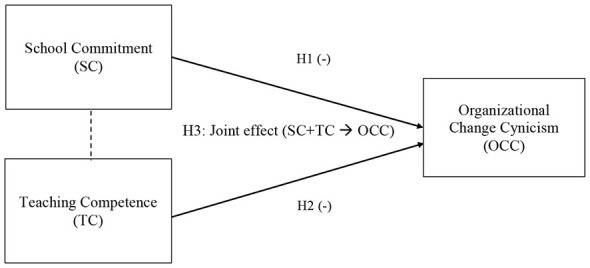
Conceptual model. H1 and H2 represent the individual predictive paths of school commitment and teaching competence to organizational change cynicism. H3 refers to the joint predictive contribution of school commitment and teaching competence to organizational change cynicism.

The conceptual model of the study is presented in Figure 1. As shown in Figure 1, the model includes the individual predictive paths of school commitment and teaching competence on organizational change cynicism (H1 and H2). H3 does not represent an additional structural path but rather refers to the joint predictive contribution of school commitment and teaching competence within a multiple regression framework. Accordingly, H3 is interpreted at the model level rather than as a separate directional relationship.

## Methodology

### Research design

This study employed a cross-sectional correlational research design to examine the relationships and predictive effects of the subdimensions of teachers' subjective well-being on their perceptions of organizational change cynicism within the framework of positive psychology theory. The correlational survey model aims to determine the direction and strength of relationships among two or more variables and to statistically examine tendencies for variables to change together (Creswell, 2012; Karasar, 2020). According to Fraenkel et al. (2012), this model functions to describe human behavior and predict potential outcomes. Consequently, this design highlights the existing relationships between variables without asserting causality. Accordingly, the findings of this study should be interpreted as associations rather than causal relationships.

### Participants

The population of this study comprises all teachers working in official schools and institutions affiliated with the Ministry of National Education in Kirşehir province as of the 2024–2025 academic year. According to the data published by the Kirşehir Provincial Directorate of National Education on February 21, 2025, there are 3977 teachers in the province. Of the 3,977 teachers constituting the population, 2,143 were reached via email and WhatsApp, and 512 teachers voluntarily participated. This figure provides a current and verifiable statistical basis for the population of the research. The sample was created by volunteer teachers in schools that could be reached through convenience sampling. Five hundred and twelve teachers working in schools in Kirşehir province were included in the research using the convenience sampling method. In the convenience sampling method, the researcher begins to create the sample, starting with the most accessible respondents until a group of the required size is reached, or works with the most accessible and cost-effective case sample (Cohen et al., 2018). With this method, the aim was to generalize teachers' opinions and evaluations in Kirşehir more reliably. The sample size was determined using the calculations of Cohen et al. (2018) based on a 95% confidence level and a 5% margin of error. The sample size of 512 participants obtained in the study provides strong representation. Descriptive findings regarding the participants are presented in Table 1.

**Table 1:** Descriptive information of participants.

Variables	Category	*f*	%
Gender	Female	343	67.0
	Male	169	33.0
Marital status	Single	105	20.5
	Married	407	79.5
Education level	College	8	1.6
	Bachelor's degree	407	79.5
	Master's degree with thesis	58	11.3
	Master's degree without thesis	35	6.8
	Doctorate	4	0.8
Professional seniority	1–5 years	41	8.0
	6–10 years	88	17.2
	11–15 years	112	21.9
	16–20 years	127	24.8
	21–25 years	86	16.8
	26 above years	58	11.3
Working time at current school	1–5 years	275	53.7
	6–10 years	102	19.9
	11–15 years	75	14.6
	16–20 years	30	5.9
	21–25 years	11	2.1
	26 above years	19	3.7

### Data collection procedure

The research data were collected online via Google Form between May 1 and June 1, 2025. Online data collection was preferred to ensure secure and easy access for participants. Before the process, a comprehensive literature review was conducted to identify the key constructs and to guide the selection of validated measurement instruments assessing subjective well-being and organizational change cynicism (Diener, 1984; Dean et al., 1998). Moreover, common method bias (CMB) is tested in the study. This procedure was undertaken because several scholars have suggested that cross-sectional survey research using self-report questionnaires, such as those employed in this study, may be susceptible to CMB (Siregar et al., 2026). Bastian and Widodo (2024) highlight that a source of error in CMB measurement is the divergence between the apparent association and the true correlation due to common method variance (CMV). Statistically, to determine whether CMV was present in the study, the Harman single-factor test was applied. This test evaluates whether a significant portion of the variance can be explained by a single factor (Agarwal and Prasad, 1998). In the analysis, all items in the questionnaire were subjected to exploratory factor analysis, and the unrotated solution was examined. According to the results, the first factor explained 44.96% of the total variance. Since this calculated value is below the 50% threshold, it can be said that common method variance does not pose a serious threat to the research findings.

### Measures

In this study, the following instruments were used as data collection tools: a personal information form, the “Teacher Subjective Well-being Questionnaire (TSWQ)”, and “Cynicism about Organisational Change Scale (COCS)”.

#### The teacher subjective wellbeing questionnaire

Teacher subjective well-being was measured using the Teacher Subjective Well-Being Questionnaire (TSWQ), originally developed by Renshaw et al. (2015). This concept is theoretically rooted in the broader literature on subjective well-being, which defines well-being as individuals' cognitive and emotional evaluations of their lives (Diener, 1984; Diener et al., 1999). TSWQ is a four-point Likert-type instrument and an 8-item scale that consists of two dimensions. In the scale, the “school commitment” dimension includes 4 items (e.g., “I feel like I belong at this school”) and the “teaching competence” dimension also includes 4 items (e.g., “I am a successful teacher”). Given their conceptual distinctiveness, these two dimensions were treated as separate variables in the present study to capture their unique contribution and to allow a more differentiated examination of their predictive roles in relation to organizational change cynicism.

The Turkish adaptation of the scale was conducted by Ergün and Sezgin Nartgün (2017), who confirmed that the instrument retains its original two-factor structure and demonstrated satisfactory psychometric properties in the Turkish educational context. In the adaptation study, internal consistency coefficients were reported as acceptable (α = 0.82 for the overall scale, α = 0.82 for the school commitment, and α = 0.79 for teaching competence). According to the CFA, the scale demonstrated good model fit (χ^2^/df = 3.11, NFI = 0.97, NNFI = 0.96, CFI = 0.98, GFI = 0.96, AGFI = 0.93, RMSEA = 0.07, SRMR = 0.04), supporting the factorial validity of the scale.

In this study, the scale also exhibited good internal consistency (α = 0.86 for the overall scale, α = 0.73 for the school commitment, and α = 0.79 for teaching competence). Also, CFA results confirmed an acceptable model fit (χ^2^/df = 3.54, CFI = 0.974, NFI = 0.964, TLI = 0.960, AGFI = 0.941, RMSEA = 0.071, SRMR = 0.0369), providing further evidence that the two-dimensional structure was retained in the current sample.

#### Cynicism about organizational change scale

Organizational change cynicism was measured using COCS, originally developed by Wanous et al. (2000). Organizational cynicism is defined as a negative attitude toward the organization characterized by beliefs of lack of integrity, negative affect, and disparaging behavioral tendencies (Dean et al., 1998). More specifically, change-related cynicism refers to pessimistic expectations regarding the success of organizational change efforts and skepticism toward change agents (Wanous et al., 2000). The COCS consists of 8 items in 2 dimensions. The “pessimism” dimension of the scale has 4 items (e.g., “The solutions provided for problems at my school are not very effective”), and the “tendency to blame the manager” dimension also has 4 items (e.g., “The administrators at my school do not do enough to address the problems”). Responses are rated on a 5-point Likert scale ranging from “strongly disagree (1) to strongly agree (5)” and do not include any reverse-coded items. The Turkish adaptation was conducted by Tülübaş and Göktürk (2021), who confirmed its two-factor structure and demonstrated strong psychometric properties in educational settings. In the adaptation study, reliability coefficients were high (α = 0.89 for pessimism; α = 0.91 for the tendency to blame the manager; α = 0.94 for the overall scale). CFA results also indicated excellent model fit (χ2/df = 1.95; NFI = 0.99, NNFI = 0.99, RMSEA = 0,040, CFI = 1,00, GFI = 0.98), supporting construct validity.

In the present study, the scale also exhibited excellent internal consistency (α = 0.950 for the overall scale, α = 0.908 for pessimism, α = 0.961 for tendency to blame the manager). CFA results confirmed an excellent model fit (χ^2^/df = 2.53, CFI = 0.995, NFI = 0.991, TLI = 0.992, IFI = 0.995, RMSEA = 0.051, SRMR = 0.015), indicating that the original factor structure was retained in the current sample.

### Data analysis

Data analysis began with validity and reliability analysis at the instrument testing stage. Common method bias (CMB) was examined, followed by descriptive and correlational analyses. Prior to hypothesis testing, the assumptions of multiple linear regression were assessed to determine whether the data were suitable for parametric analysis. All analyses were performed using IBM SPSS Statistics 25, and CFA was performed using AMOS to evaluate the construct validity of the measurement instruments. To examine the predictive relationships among the study variables, a multiple linear regression analysis was conducted. School commitment and teaching competence were entered as predictor variables, while organizational change cynicism was treated as the dependent variable. Although the measurement model included multiple subdimensions of organizational change cynicism, a composite score was used in the regression analysis to ensure model parsimony and to reduce potential multicollinearity among closely related constructs. This approach is consistent with prior research suggesting that highly correlated subdimensions may introduce estimation bias when included simultaneously in regression models. Also, the data did not include school-level identifiers; therefore, clustering effects could not be directly tested.

#### Measurement model and psychometric properties

To examine the construct validity of the measurement instruments used in the study, Composite Reliability (CR) and Average Variance Extracted (AVE) values were used for convergent validity, and Heterotrait-Monotrait (HTMT) correlation ratio values and the Fornell-Larcker criterion were used for discriminant validity (Table 2).

**Table 2:** Assessment of measurement quality: CR, AVE, Fornell-Larcker, and HTMT.

Subscales	CR	AVE	HTMT ratio				Fornell-Larcker criterion			
							1	2	3	4
1. School commitment (SC)	0.809	0.520	—				0.721			
2. Teaching competence (TC)	0.839	0.566	0.85	—			0.547	0.752		
3. Pessimism (P)	0.912	0.724	0.61	0.54	—		−0.490	−0.197	0.851	
4. Tendency to blame the manager (TBM)	0.964	0.869	0.28	0.22	0.62	—	−0.433	−0.142	0.769	0.932

To establish convergent validity, the Average Variance Extracted (AVE) should be above 0.50, and the Composite Reliability (CR) should exceed 0.70 (Fornell and Larcker, 1981; Hair et al., 2019). Both scales, as illustrated in Table 2, exhibit Composite Reliability (CR) values above 0.70, with values surpassing 0.80. Additionally, their Average Variance Extracted (AVE) values exceed 0.52, which is above the required minimum threshold. These results indicate that the scales have achieved convergent validity and demonstrate high internal consistency. Additionally, Cronbach's alpha coefficients were analyzed, with all values exceeding 0.70, indicating good reliability. The factor loadings for both scales were also satisfactory, ranging from 0.556 to 0.844 for SWB and from 0.725 to 0.941 for OCC. Overall, these findings provide strong support for the reliability of the measurement instruments (Hair et al., 2019).

Discriminant validity measures the degree to which a construct differs empirically from other constructs, as well as the level of difference between overlapping constructs (Ab-Hamid et al., 2017). Discriminant validity was first assessed using the Fornell-Larcker criterion. According to this criterion, the square root of the AVE score must be greater than the correlation coefficient with other variables (Fornell and Larcker, 1981).

The findings in Table 2 indicate that discriminant validity has been achieved. The HTMT correlation ratio is also evaluated in the control of discriminant validity. HTMT values of 0.85 and below are known to indicate strong discrimination; values below 0.90 are also acceptable (Henseler et al., 2015). Although the correlation between pessimism and the tendency to blame the manager is relatively high, it does not exceed commonly accepted threshold values. The findings support the establishment of discriminant validity between the sub-dimensions of both scales according to the HTMT values. Additionally, discriminant validity is supported by both the Fornell-Larcker criterion and the HTMT ratio.

#### Assumptions of regression analyses

Before the main analysis assumptions underlying Pearson correlation and multiple regression were examined in detail. The distribution of the observed variables was initially assessed using skewness and kurtosis values. All values were within the range of ±1.5, and the measures of central tendency were close, indicating that the data follow a normal distribution (Field, 2018; Tabachnick and Fidell, 2013). Greater emphasis was placed on the assumptions related to regression residuals, as these are more critical for statistical inference (Field, 2018; Tabachnick and Fidell, 2013). Linearity between independent and dependent variables was evaluated using scatterplots of standardized predicted values and residuals, which indicated approximately linear relationships. The same plots were also used to evaluate the assumptions of homoscedasticity, and the distribution of residuals suggested that the variance was relatively constant across levels of predicted values, supporting the assumption of homoscedasticity (Field, 2018). The normality of residuals was assessed using standardized residual statistics. The standardized residuals ranged between −2.48 and 3.73, with a mean close to zero and a standard deviation approximately equal to one, indicating that the residuals were approximately normally distributed.

Multicollinearity was evaluated using the Variance Inflation Factor (VIF) values as well as collinearity diagnostics. including eigenvalues. condition indices. and variance proportions. The VIF values were low (1.426), remaining well below the critical value of 5, indicating no concerns regarding multicollinearity (Hair et al., 2019; Tabachnick and Fidell, 2013). Additionally, the maximum condition index (CI) was 16.688, which is below the critical value of 30 and the variance proportions did not suggest any problematic multicollinearity (Belsley et al., 1980). Although one predictor exhibited a high variance proportion in a single dimension. No other predictor showed similarly high values within the same dimension, suggesting that multicollinearity was not a problem (Field, 2018). Potential outliers and influential observations were examined using standardized residuals. Mahalanobis distance and Cook's distance values. Standardized residuals within ±3. Cook's distance values (max = 0.048) below 1, and acceptable Mahalanobis distance values indicated that no influential cases or multivariate outliers were present (Cohen et al., 2003; Hair et al., 2019). Standardized residual values remained within acceptable limits. further indicating the absence of problematic outliers. Finally, the independence of errors was evaluated using the Durbin-Watson statistic. Although values within the range of 1.5–2.5 are generally considered acceptable (Kutner et al., 2005). This statistic is primarily appropriate for time-series data and should be interpreted with caution in cross-sectional studies (Field, 2018). Overall, these findings indicate that the assumptions required for multiple regression were adequately met. and the results can be considered robust.

### Ethics approval and consent to participate

This study was conducted in accordance with the original version of the Declaration of Helsinki, adopted in 1964, as well as its subsequent updates. Before the study started, the necessary ethical approval was obtained from Kirşehir University Ethics Committee (Ethics Committee Approval Number: 2025-12-07/13.08.2025). Participants were informed about the research's purpose, process, and principles of voluntary participation. The confidentiality of participants and their data was meticulously protected.

### Findings

This section presents the findings of the study, including descriptive statistics, correlation analysis, and multiple linear regression results.

### Descriptive and correlational analysis

The correlation coefficients, arithmetic mean, standard deviation, skewness, and kurtosis for teachers' scores on organizational change cynicism, school commitment, and teaching competence are summarized in Table 3.

**Table 3:** Statistics on variables.

Variables	*n*	$\bar{X}$	SD	Skewness	Kurtosis	1	2	3
1. School commitment (SC)	512	3.15	0.67	−0.49	−0.63	1		
2. Teaching competence (TC)	512	3.38	0.52	−0.56	−0.34	0.547^**^	1	
3. Organizational change cynicism (OCC)	512	2.26	0.93	0.76	0.23	−0.487^**^	−0.177^**^	1

^**^p < 0.01.

As seen in Table 3, the mean scores (ranging from 2.26 to 3.38) exceeded the corresponding standard deviation values (0.52 and 0.93). This reflects a suitable data representation and deserves further analysis. Meanwhile, correlation analysis showed that all indicators had a significant relationship with the others at *p* < 0.01, with a correlation coefficient range of −0.487 to 0.547; these findings suggest the presence of meaningful interrelationships among the variables. Importantly, none of the observed correlations approached the critical threshold of 0.80, indicating that multicollinearity is not a concern in the dataset (Bastian and Widodo, 2024).

A multiple linear regression analysis was conducted to examine the extent to which school commitment and teaching competence predict teachers' organizational change cynicism, and the results are presented in Table 4. Although the measurement model included subdimensions of organizational change cynicism, a composite score was used in the regression analysis to ensure parsimony and to reduce potential multicollinearity among closely related constructs.

**Table 4:** Multiple linear regression analysis results.

Predictor	B	SE	β	t	*p*	Tolerance	VIF
Constant	3.926	0.238	—	16.476	< 0.001	—	—
School commitment (SC)	−0.770	0.063	−0.557	−12.132	< 0.001	0.701	1.426
Teaching competence (TC)	0.227	0.081	0.128	2.784	0.006	0.701	1.426

*N* = 512. *R* = 0.499, *R*^2^ = 0.249, Adjusted *R*^2^ = 0.246, F_(2, 509)_ = 84.168 Dependent variable = Organizational Change Cynicism. Unstandardized (B) and standardized (β) coefficients are reported.

According to Table 4, the results indicated that the overall regression model was statistically significant (*R* = 0.499, *R*^2^ = 0.249, Adj. *R*^2^ = 0.246, F_(2, 509)_ = 84.168, *p* < 0.001), explaining 24.9% of the variance in organizational change cynicism. Regarding the hypotheses, **H1** proposed that school commitment negatively predicts organizational change cynicism, and this hypothesis was fully supported. School commitment was found to be a strong and significant negative predictor (β = −0.557, *t* = –12.132, *p* < 0.001).

**H2** proposed that teaching competence negatively predicts organizational change cynicism, but this was not supported as expected. Although teaching competence was statistically significant (β = 0.128, *t* = 2.784, *p* = 0.006), its positive coefficient contradicted the hypothesized negative relationship. This unexpected finding suggests the presence of a suppression effect, indicating that teaching competence shares variance with school commitment and reveals its unique effect only after controlling for this shared variance (Conger, 1974; Friedman and Wall, 2005; MacKinnon et al., 2000).

**H3** proposed that school commitment and teaching competence jointly predict organizational change cynicism, and this hypothesis was fully supported as well. The model was statistically significant, confirming the combined predictive power of the two variables.

Multicollinearity diagnostics confirmed that the regression assumptions were met (Tolerance = 0.701; VIF = 1.426), indicating no concerns regarding multicollinearity. Additionally, the Durbin-Watson statistic (1.809) showed that there was no autocorrelation in the residuals.

## Discussion

The present study examined the extent to which school commitment and teaching competence predict teachers' organizational change cynicism. The findings revealed three key results. First, school commitment emerged as a strong and significant negative predictor of organizational change cynicism. Second, teaching competence, although weakly and negatively associated with cynicism at the bivariate level, demonstrated a positive and significant effect in the regression model. Third, the two variables jointly explained a meaningful proportion of variance in organizational change cynicism. Taken together, these findings suggest that teachers' responses to organizational change are shaped by both their relational attachment to the school and their professional evaluations of instructional and organizational processes.

The finding that school commitment negatively predicts organizational change cynicism is consistent with the broader organizational literature emphasizing the role of psychological attachment in shaping employees' attitudes and behaviors (Meyer and Allen, 1991; Meyer et al., 2002). This interpretation is also compatible with prior evidence linking cynicism to reduced commitment and stronger resistance to change (Toheed et al., 2019). Commitment has been associated with stronger identification with organizational goals, higher levels of engagement, and more constructive responses to organizational demands (Firestone and Pennell, 1993; Somech and Bogler, 2002). In change contexts, employees with higher commitment tend to demonstrate more supportive attitudes toward organizational initiatives and lower tendencies toward withdrawal or resistance (Oreg et al., 2011; Vakola, 2014). In educational settings, similar patterns have been observed, with committed teachers showing more stable professional engagement and lower tendencies toward disengagement and withdrawal-related attitudes (Skaalvik and Skaalvik, 2011). In this respect, the present findings suggest that school commitment may function as a relational factor associated with less negative and more constructive interpretations of organizational change processes.

The results regarding teaching competence require a more careful interpretation. While competence is widely associated with positive professional outcomes such as instructional effectiveness and teacher self-efficacy (Shulman, 1987; Tschannen-Moran and Hoy, 2001; Darling-Hammond, 2017; Blömeke et al., 2015), the present findings indicate that its relationship with organizational change cynicism is not straightforward. Although teaching competence was negatively associated with cynicism at the descriptive level, its positive regression coefficient suggests that its unique contribution becomes visible only when its shared variance with school commitment is controlled. This pattern is consistent with the statistical notion of a suppression effect, whereby the inclusion of a related predictor clarifies or alters the relationship between another predictor and the outcome variable (Conger, 1974; MacKinnon et al., 2000; Friedman and Wall, 2005).

This finding extends the existing literature by suggesting that teaching competence may operate in more complex ways than commonly assumed. On the one hand, competence supports effective instructional practices and professional confidence. On the other hand, research on teacher professionalism indicates that more competent teachers tend to develop stronger professional judgment and greater sensitivity to instructional quality and organizational practices (Kelchtermans, 2009; Priestley et al., 2015). As a result, they may be more likely to critically evaluate organizational change initiatives, particularly when such initiatives are perceived as insufficiently aligned with pedagogical standards or classroom realities.

From this perspective, the positive association between teaching competence and organizational change cynicism observed in the regression model should not be interpreted as a simple form of resistance. Rather, it may reflect a form of professionally grounded critical evaluation. Teachers with higher competence may be more capable of identifying limitations in the design, implementation, or relevance of change initiatives and may therefore adopt a more questioning stance toward such processes. This interpretation suggests that, under certain conditions, what appears as cynicism may partly reflect a critical orientation grounded in professional standards rather than a purely negative or disengaged attitude.

The coexistence of a strong negative effect of school commitment and a weaker positive effect of teaching competence highlights the importance of distinguishing between relational and professional dimensions of teachers' organizational experiences. While commitment appears to anchor teachers to the organization and foster more supportive attitudes, competence may encourage evaluative thinking and critical appraisal. When these dimensions are considered simultaneously, they may produce patterns that are not evident in simple bivariate relationships. This finding underscores the importance of distinguishing between relational attachment and professional evaluation when examining attitudes toward organizational change.

The study contributes to the literature in several ways. First, it extends research on organizational change cynicism by demonstrating that teachers' attitudes are shaped not only by organizational conditions but also by the interplay between organizational attachment and professional competence. Second, it highlights that teaching competence should not be viewed solely as a uniformly positive resource but also as a factor that may be associated with more critical evaluations under certain conditions. Third, the identification of a suppression pattern provides a methodological contribution by illustrating how relationships among predictors may change when shared variance is taken into account.

From a practical perspective, the findings suggest that efforts to reduce organizational change cynicism in schools should not rely solely on structural or administrative interventions. Strengthening teachers' commitment to their schools may be associated with more constructive attitudes toward change processes. At the same time, the critical perspectives of highly competent teachers should not be interpreted solely as resistance, but also as potentially informative professional feedback, but also as potentially informative professional feedback. Instead, such perspectives may provide valuable feedback regarding the quality, relevance, and implementation of reform initiatives, and may contribute to more meaningful and sustainable change processes.

Finally, the findings should be interpreted within the limitations of a cross-sectional and correlational research design. The observed relationships do not imply causality, and future research could benefit from longitudinal or mixed-method approaches to better understand how teachers' commitment and competence interact over time in shaping responses to organizational change.

## Conclusion

This study investigated the extent to which school commitment and teaching competence predict teachers' organizational change cynicism. The findings demonstrated that school commitment is a strong negative predictor of organizational change cynicism, whereas teaching competence exhibits a more complex relationship, revealing a positive association when examined alongside school commitment. Together, these variables explain a meaningful proportion of variance in teachers' attitudes toward organizational change.

These results highlight that teachers' responses to change are shaped by both relational and professional dimensions of their work. School commitment, as an indicator of teachers' psychological attachment to their institutions, appears to be associated with more constructive interpretations of change processes. In contrast, teaching competence, while generally considered a professional strength, may, under certain organizational conditions, be associated with more critical evaluations of organizational change under certain conditions. This finding suggests that competence does not operate solely as a uniformly positive resource but may also reflect a more evaluative orientation toward institutional practices.

The study contributes to the literature in two important ways. First, it provides evidence that organizational change cynicism in educational settings is influenced not only by contextual and organizational factors but also by the interplay between teachers' attachment to the school and their professional competence. Second, it highlights the importance of considering potential suppression effects in examining relationships among predictors, as such effects may reveal patterns that are not apparent in bivariate analyses.

From a practical perspective, the findings suggest that efforts to support successful organizational change in schools should address both relational and professional dimensions. Strengthening teachers' commitment to their schools may be associated with more supportive attitudes toward change initiatives. At the same time, the critical perspectives of highly competent teachers should be recognized not only as potential resistance but also as valuable sources of professional insight sources of professional insight rather than being interpreted solely as resistance. Engaging with these perspectives may contribute to more context-sensitive and sustainable reform processes.

Finally, several limitations should be acknowledged. The cross-sectional design of the study limits the ability to draw causal conclusions, and the reliance on self-reported data may introduce response bias. Future research could benefit from longitudinal designs and mixed-method approaches to better understand how teachers' commitment and competence interact over time in shaping their responses to organizational change. Additionally, examining these relationships across different educational contexts may provide further insight into the generalizability of the findings.

### Theoretical implications

The findings of this study offer several important contributions to the literature on organizational change and educational organizations.

First, the study contributes to the literature on organizational change cynicism by suggesting that teachers' attitudes toward change may be shaped not only by organizational and contextual factors but also by the interplay between individual-level relational and professional variables. While prior research has primarily focused on organizational antecedents such as leadership, communication, and justice perceptions (Oreg et al., 2011; Vakola, 2014), the present findings indicate that teachers' psychological attachment to the school and their professional competence may also serve as relevant explanatory factors. This highlights the value of integrating organizational and professional perspectives in understanding attitudes toward change in educational settings.

Second, the study provides a more nuanced understanding of school commitment by positioning it as a relational factor that may be associated with more constructive interpretations of organizational processes. Consistent with organizational commitment theory (Meyer and Allen, 1991; Meyer et al., 2002), the findings support the view that commitment is not only an attitudinal outcome but may also shape how individuals interpret and respond to organizational events. In the context of educational change, this suggests that commitment may be associated with reduced tendencies toward negative interpretations of reform processes.

Third, the study extends the literature on teaching competence by indicating that its role in shaping organizational attitudes may be more complex than traditionally assumed. This interpretation is compatible with broader views of competence as an integrated professional construct involving knowledge, skills, motivations, and attitudes, rather than a purely technical resource (Mara and Morar, 2023). Although competence is commonly conceptualized as a positive professional resource associated with effective teaching and adaptive functioning (Shulman, 1987; Tschannen-Moran and Hoy, 2001), the present findings suggest that it may also be associated with more critical evaluations of organizational change under certain conditions. This interpretation is broadly aligned with perspectives in teacher professionalism that emphasize the role of professional judgment and reflective practice (Kelchtermans, 2009; Priestley et al., 2015). In this sense, the study highlights the possibility that competence may involve both resource-based and evaluative dimensions of professional practice.

Fourth, the study makes a methodological contribution by illustrating the potential relevance of suppression effects in educational research. This argument is also strengthened by recent work suggesting that cynicism about organizational change may involve multiple dimensions with distinct outcome patterns (Gupta and Mishra, 2024). The finding that teaching competence exhibits a positive association with organizational change cynicism when school commitment is controlled, despite a negative bivariate relationship, suggests that relationships among predictors may change when shared variance is taken into account (Conger, 1974; MacKinnon et al., 2000; Friedman and Wall, 2005). This underscores the importance of examining multivariate relationships rather than relying solely on zero-order correlations when interpreting complex organizational phenomena.

Finally, the study advances the literature by emphasizing the distinction between relational attachment and professional evaluation as two complementary but potentially divergent dimensions of teachers' organizational experiences. While school commitment reflects teachers' emotional and organizational ties, teaching competence reflects their professional judgment and evaluative capacity. The findings suggest that these dimensions may operate through different psychological mechanisms and may lead to different interpretations of organizational change. This distinction may provide a useful conceptual lens for future research seeking to better understand the complexity of teachers' attitudes toward change in educational settings.

### Practical implications

The findings of this study offer several practical implications for school leaders, policymakers, and other stakeholders involved in educational change processes.

First, the strong negative association between school commitment and organizational change cynicism indicates that strengthening teachers' sense of attachment to their schools is likely to support more constructive attitudes toward change initiatives. School leaders may therefore benefit from fostering a supportive and inclusive organizational climate in which teachers feel valued, respected, and connected to institutional goals. Practices such as participatory decision-making, transparent communication, and recognition of teachers' contributions can help reinforce this sense of commitment and promote more positive responses to organizational change.

Second, the findings suggest that teaching competence, while generally considered a professional strength, may also be associated with more critical evaluations of change processes under certain conditions. This indicates that the perspectives of highly competent teachers should not be interpreted solely as resistance to change, but also as potentially valuable professional input. School leaders may therefore benefit from actively involving competent teachers in the design, evaluation, and refinement of change initiatives, rather than excluding or marginalizing their critical viewpoints.

Third, the combined effects of school commitment and teaching competence highlight the importance of adopting a balanced and context-sensitive approach to managing organizational change. Efforts that focus exclusively on increasing compliance or minimizing resistance may overlook the constructive role of professional critique. Change processes are likely to be more effective when they simultaneously support teachers' organizational attachment and create opportunities for professional dialogue and reflection. Encouraging collaborative environments in which teachers can openly discuss, question, and refine change initiatives may contribute to more sustainable and context-responsive reforms.

Finally, the identification of a potential suppression effect suggests that school leaders and policymakers may need to be cautious when interpreting teachers' attitudes toward change. Such attitudes may not always reflect simple acceptance or resistance; rather, they may involve a combination of relational attachment and professional evaluation. Recognizing this complexity may help stakeholders develop more nuanced and context-responsive strategies for implementing and sustaining organizational change in educational settings.

## Limitations and recommendations

While this study provides important insights into the relationships between school commitment, teaching competence, and organizational change cynicism, several methodological considerations should be taken into account when interpreting the findings.

The data were collected exclusively from teachers working in Kirşehir, Türkiye, which introduces a contextual limitation. Educational environments vary substantially in terms of socio-cultural structures, institutional practices, and leadership dynamics. Such differences may influence both teachers' organizational attachment and their perceptions of change processes. For this reason, the findings are most appropriately interpreted within contexts that share similar characteristics, and caution is warranted when extending them to broader or structurally different educational systems.

The sampling strategy also presents a limitation in terms of external validity. The use of an accessibility-based, non-probability sampling approach was shaped by practical constraints, including access permissions, time limitations, and data collection conditions. Although such constraints are common in educational research, they may restrict the representativeness of the sample. As a result, the findings should be considered with an awareness of their contextual boundaries.

The cross-sectional and self-report-based nature of the research design further limits the ability to draw causal conclusions. Since all variables were measured at a single point in time and obtained from the same source, the results may be influenced by common method variance (CMV). Although procedural and statistical steps were taken to reduce this risk, it cannot be entirely ruled out and should be taken into consideration when interpreting the relationships among variables.

The exclusive reliance on quantitative data also constrains the depth of interpretation. Teachers' responses to organizational change are shaped by complex emotional, contextual, and experiential factors that may not be fully captured through standardized measurement tools. In addition, the absence of school-level variables limits the ability to account for potential clustering effects. While the analyses were conducted at the individual level, unobserved institutional influences may have affected the relationships among variables. Approaches such as multi-level modeling could provide a more comprehensive understanding of these nested dynamics in future research.

Another point requiring careful consideration is the potential presence of a suppression effect, which indicates that relationships among variables may not be fully captured through bivariate associations alone. This suggests that the observed patterns should be interpreted with caution and highlights the importance of examining multivariate relationships in greater detail.

These limitations point to several directions for future research. Expanding the scope of the sample to include teachers from diverse regions and educational contexts would strengthen the generalizability of the findings and allow for more robust cross-context comparisons. Longitudinal and experimental designs would be particularly valuable in clarifying the causal dynamics among school commitment, teaching competence, and organizational change cynicism, as well as in identifying potential reciprocal relationships.

Further research may also benefit from incorporating additional variables into more comprehensive models. Constructs such as organizational justice, leadership styles, job satisfaction, and professional collaboration may play important mediating or moderating roles in shaping teachers' responses to change processes. Exploring these relationships could contribute to a more holistic understanding of organizational change in educational settings.

The use of qualitative or mixed-methods approaches represents another promising direction. Such approaches could provide deeper insight into how teachers interpret and experience change, particularly in relation to the role of professional competence in shaping critical evaluations of reform initiatives. This would help distinguish between resistance, professional judgment, and context-specific concerns.

From a practical perspective, future studies may also examine the effectiveness of organizational and professional development interventions aimed at strengthening school commitment and supporting constructive professional engagement. Understanding how such interventions influence teachers' attitudes toward change may contribute to the development of more sustainable and context-responsive reform practices in educational institutions.

## Data Availability

The original contributions presented in the study are included in the article/supplementary material, further inquiries can be directed to the corresponding author.
